# N-myc Downstream Regulated 1 (NDRG1) Is Regulated by Eukaryotic Initiation Factor 3a (eIF3a) during Cellular Stress Caused by Iron Depletion

**DOI:** 10.1371/journal.pone.0057273

**Published:** 2013-02-21

**Authors:** Darius J. R. Lane, Federica Saletta, Yohan Suryo Rahmanto, Zaklina Kovacevic, Des R. Richardson

**Affiliations:** Molecular Pharmacology and Pathology Program, Department of Pathology and Bosch Institute, University of Sydney, Sydney, New South Wales, Australia; Indiana University School of Medicine, United States Of Ameica

## Abstract

Iron is critical for cellular proliferation and its depletion leads to a suppression of both DNA synthesis and global translation. These observations suggest that iron depletion may trigger a cellular “stress response”. A canonical response of cells to stress is the formation of stress granules, which are dynamic cytoplasmic aggregates containing stalled pre-initiation complexes that function as mRNA triage centers. By differentially prioritizing mRNA translation, stress granules allow for the continued and selective translation of stress response proteins. Although the multi-subunit eukaryotic initiation factor 3 (eIF3) is required for translation initiation, its largest subunit, eIF3a, may not be essential for this activity. Instead, eIF3a is a vital constituent of stress granules and appears to act, in part, by differentially regulating specific mRNAs during iron depletion. Considering this, we investigated eIF3a’s role in modulating iron-regulated genes/proteins that are critically involved in proliferation and metastasis. In this study, eIF3a was down-regulated and recruited into stress granules by iron depletion as well as by the classical stress-inducers, hypoxia and tunicamycin. Iron depletion also increased expression of the metastasis suppressor, N-myc downstream regulated gene-1 (NDRG1), and a known downstream repressed target of eIF3a, namely the cyclin-dependent kinase inhibitor, p27^kip1^. To determine if eIF3a regulates NDRG1 expression, eIF3a was inducibly over-expressed or ablated. Importantly, eIF3a positively regulated NDRG1 expression and negatively regulated p27^kip1^ expression during iron depletion. This activity of eIF3a could be due to its recruitment to stress granules and/or its ability to differentially regulate mRNA translation during cellular stress. Additionally, eIF3a positively regulated proliferation, but negatively regulated cell motility and invasion, which may be due to the eIF3a-dependent changes in expression of NDRG1 and p27^kip1^ observed under these conditions.

## Introduction

Translation initiation is the major rate-limiting step in translation and involves many eukaryotic initiation factors (eIFs) [1]. Among the eIF family, eIF3 is the largest and most complex initiation factor and contains 13 different subunits designated eIF3a-m [2], [3]. The largest subunit of eIF3 is eIF3a (also known as p170) [3]. Importantly, while *in vitro* reconstitution analyses indicate eIF3a may be a core eIF3 subunit involved in translation initiation [4], other studies suggest this protein may not be essential for the general function of eIF3 in global translation initiation, and/or it may have auxiliary functions [3], [5]. This latter claim is supported by the following observations: ***(i)*** reticulocyte eIF3 preparations with or without eIF3a do not significantly differ in their translation initiation activity [5]; ***(ii)*** marked depletion of eIF3a by anti-sense constructs suppresses global translation by only 15–20% [6], [7]; and ***(iii)*** 20S proteasomal degradation of eIF3a affects the assembly of ribosomal pre-initiation complexes only on defined, but not global mRNA populations [8].

Interestingly, eIF3a differentially regulates translation of a defined subset of mRNAs in the presence of the iron chelator, l-mimosine [7]. Indeed, the ability of l-mimosine to slow progression through the cell cycle [7], [9] depends, at least in part, on the activity of eIF3a as a transcript-specific translational regulator [6], [7]. Incubation of cells with l-mimosine decreases eIF3a expression and causes an eIF3a-dependent: ***(i)*** increase in translation of the cyclin-dependent kinase inhibitor, p27^kip1^ and ***(ii)*** a decrease in translation of tyrosinated *α*-tubulin and the ribonucleotide reductase M2 subunit [6], [7]. Thus, eIF3a differentially regulates a subset of transcripts encoding proteins (*e.g.*, p27^kip1^ and ribonucleotide reductase M2) involved in cell cycle progression and cell growth [10]–[12]. Additionally, eIF3a functions as a translational regulator for several key nucleotide excision repair proteins [13]. Taken together, these properties of eIF3a may explain observations that eIF3a over-expression occurs in cancer [3], [14]. Further, eIF3a depletion reduces key malignant attributes such as proliferation [6], while it increases resistance to treatment with DNA damaging agents [13].

As l-mimosine suppresses global translation as a probable consequence of its iron-chelating activity [7], the l-mimosine-mediated decrease in eIF3a expression is likely to be reflective of the cellular “stress response”. When the latter is activated, global translation is typically suppressed, while certain transcripts encoding key stress-response proteins continue to be translated [15]. While the mechanism by which eIF3a selectively regulates the translation of specific transcripts is unknown, this activity could be a consequence of eIF3a’s role as a constituent of stress granules [16], [17]. Stress granules are cytoplasmic structures that form after exposure to stressors, such as heat shock, oxidative stress, hypoxia *etc* [18]. They are composed of stalled pre-initiation complexes, small ribosomal subunits and initiation factors (*e.g.*, eIF3) [18]. Indeed, the stress granule acts as a “triage center” that sorts, remodels and/or exports specific mRNAs for decay, storage or translation [19]. This process enables translation of only those proteins that are vital for the stress response [20]. Hence, eIF3a may modulate translation of specific transcripts during stress by regulating their distribution between stress granules, polysomes and the sites of mRNA decay [15], [20], [21].

It has been demonstrated that the well characterized metastasis suppressor, N-myc downstream regulated 1 (NDRG1) [22], is up-regulated at the levels of both mRNA and protein after treatment with l-mimosine [23]. This suggests that eIF3a may play a role in regulating NDRG1 expression, although this hypothesis has never been examined. Intriguingly, *NDRG1* has been identified as a stress-response gene [24], [25] and it was described as a metastasis suppressor in a wide variety of tumors [26]–[29].

*NDRG1* is also a hypoxia-regulated gene [30]–[32] and the hypoxia-inducible factor-1*α* (HIF-1*α*)-dependent up-regulation of *NDRG1* transcription has been observed [30], [33], [34]. Further, *NDRG1* is markedly induced by iron depletion mediated by the iron chelators, desferrioxamine (DFO) and 2-hydroxy-1-naphthaldehyde isonicotinoyl hydrazone (311) [35]–[37]. Iron chelators mimic hypoxia by inhibiting prolyl hydroxylation and subsequent proteasome-mediated degradation of HIF-1*α* [38]. Indeed, we showed that regulation of NDRG1 after iron depletion involves HIF-1*α*-dependent and -independent mechanisms [35].

The current investigation focused on examining the hypothesis that eIF3a contributes to regulating NDRG1 expression during iron depletion. For the first time, this study demonstrates that eIF3a is recruited to stress granules after iron depletion and that eIF3a positively regulates NDRG1 expression under these conditions. The latter event was accompanied by decreased eIF3a expression that occurs during iron depletion and is reflective of the cellular stress response. Indeed, the stress response depends on the continued translation of specific stress-response proteins in the face of suppressed global protein synthesis. To assess the role of eIF3a in NDRG1 regulation, we implemented eIF3a over-expression and complete ablation models using stably-transfected sense and anti-sense constructs. Notably, eIF3a over-expression potentiated the increase in NDRG1 by iron depletion, while anti-sense ablation of eIF3a diminished this response. These results show that eIF3a plays a role in NDRG1 expression. Hence, despite decreased eIF3a expression after iron depletion, this protein potentiates iron-regulated NDRG1 expression. In addition, ablation of eIF3a decreased proliferation, but increased cell motility and invasion, while its over-expression led to the opposite response. As these functional alterations correlated with changes in expression of NDRG1 and the eIF3a repressed target, p27^kip1^, these alterations may explain how eIF3a mediates its effects on metastasis and proliferation, respectively.

## Materials and Methods

### Reagents

The chelator, 311, was synthesized and characterized as reported [39], while DFO was from Novartis. Ferric ammonium citrate (FAC), tunicamycin, hygromycin B from *Streptomyces hygroscopicus*, tetracycline hydrochloride (TET) and 3-(4,5-dimethyl-2-thiazolyl)-2,5-diphenyl-2H-tetrazolium bromide (MTT) were from Sigma-Aldrich.

### Cell Culture

MCF7 breast cancer cells were from the American Type Culture Collection and cultured, as described [35]. MCF7 TET-Off^®^ cells were obtained from Clontech. Murine embryonic fibroblasts (MEFs) from wild-type and homozygous *HIF-1α* knockout mice were obtained from Dr. R. Johnson (University of California, San Diego, CA, USA) [40], [41]. Cells were cultured using standard conditions at 37°C in the presence of medium containing 10% fetal calf serum (FCS). Unless otherwise specified, cells were incubated under standard conditions in an atmosphere of 95% air/5% CO_2_.

In several studies, cells were incubated with the well characterized iron chelators, DFO (250 µM) or 311 (25 µM), for 24 h/37°C [39], [42]. These ligands and experimental conditions have been clearly demonstrated to induce cellular iron depletion in these and other cell-types [39], [42]–[45]. The higher concentration of DFO, relative to 311, was implemented due to its limited ability to permeate cells [42], [45], [46]. The iron-chelator, 311, was utilized at a lower concentration since this ligand shows far greater membrane permeability than DFO and demonstrates pronounced iron chelation efficacy [42], [45]. In some experiments, cells were incubated under hypoxic conditions in an atmosphere of 1% O_2_, 94% N_2_ and 5% CO_2_.

### eIF3a TET-Regulated Cells

The pC*β*A-*eIF3a* and pCMV-*eIF3a-AS* plasmids were a kind gift from Prof. Jian-Ting Zhang (Indiana University School of Medicine, IN, USA). Transfections using these plasmids were carried out with MCF7 TET-Off^®^ cells according to the manufacturer’s instructions. Briefly, *eIF3a* and *eIF3a-AS* fragments were subcloned into a pTRE2hyg/pur plasmid (Clontech) and transfected in TET-Off-MCF7 cells. Cells were then treated with hygromycin (200 µg/mL) for 2–4 weeks in order to select those transfected with the pTRE (TET-responsive element) plasmids to generate a stable transfection. Clones of hygromycin-resistant cells were obtained by limiting dilution and then tested with RT-PCR and westerns to assess expression of *eIF3a* mRNA and protein and examine the response to TET treatment.

### Immunofluorescence

Immunofluorescence was performed according to standard procedures. Briefly, cells seeded on cover slips were fixed with 4% (w/v) paraformaldehyde (Sigma-Aldrich) for 10 min and permeabilized with 0.1% Triton X-100 for 5 min at room temperature. The cells were then incubated overnight at 4°C with the following primary antibodies: rabbit polyclonal anti-human eIF3a (“eIF3θ (H300)”, Santa Cruz Biotechnology, Cat. # sc-30149, 1∶150–1∶250), rabbit polyclonal anti-human NDRG1 (Sigma-Aldrich, Cat. # HPA006881, 1∶150–1∶250) and mouse monoclonal anti-human eIF2*α* (L57A5; Cell Signaling Technology, Cat. # 2103, 1∶250). This procedure was followed by an incubation with one or both of the following secondary antibodies for 1 h at room temperature: fluorescent Alexa Fluor 555^®^ (“red”) conjugated anti-rabbit IgG (H+L), F(ab’)_2_ fragment (Cell Signaling Technology; Cat. #4413S, 1∶1000), or a fluorescent Alexa Fluor 488^®^ (“green”) conjugated anti-mouse IgG (H+L), F(ab’)_2_ fragment (Cell Signaling Technology; Cat. #4408, 1∶1,000).

After final washes with PBS, the cover-slips were mounted using an anti-fade mounting solution containing 4',6-diamidino-2-phenylindole (DAPI; Cat. P36935, Invitrogen) and images were examined and captured using an Olympus Zeiss AxioObserver Z1 fluorescence microscope (Olympus) with a 63× oil objective. A series of 30–50 optical cross-sections were taken through z (thickness) intervals of 0.33 µm. The resulting Z-stack was de-convolved implementing Zeiss AxioVision software (Olympus) using the inverse filter algorithm. The projection of the 3^rd^ dimension was used to ensure that the appropriate “slice” (through the nucleus) was observed. Evaluation of the dimensions of intracellular structures was performed using the Integrated Morphometry Analysis (IMA) module of MetaMorph^®^ Microscopy Automation and Image Analysis Software (Molecular Devices) [47]. The quantitation of eIF3a and NDRG1 expression was performed by assessing 300 cells under each condition in three different experiments. The results of this analysis are represented as signal intensity fold change.

### RNA Isolation and RT-PCR

Total RNA was isolated using TRIzol^®^ (Invitrogen). Semi-quantitative RT-PCR was performed using SuperScript III RT/Platinum^®^ Taq mix (Invitrogen). RT-PCR was shown to be semi-quantitative by an optimization protocol which demonstrated it was in the log-phase of amplification [48]. The sequences of the primers implemented are listed in Table 1. The house-keeping gene, *β-actin*, was co-amplified as an internal standard.

**Table 1:** Primers for amplification of genes used in this study.

Primer name	Genbank Accession No.	Product Size (bp)	Oligonucleotides (5′-3′)	
			Forward	Reverse
**eIF3a**	**NM_003750.2**	**313**	**ACAGGCAGTGTTTGGAC**	**GAGAATAGCCCGTGAATA**
**p27^kip1^**	**NM_004064.3**	**347**	**GCAAGTACGAGTGGCAAGA**	**CGTCTGCTCCACAGAACC**
**NDRG1**	NM_006096	**617**	TCACCCAGCACTTTGCCGTCT	GCCACAGTCCGCCATCTT
**TfR1**	NM_003234	**359**	GCTCGGCAAGTAGATGGC	TTGATGGTGCTGGTGAAG
**p53**	**NM_000546**	**422**	**ACCCAGGTCCAGATGAAG**	**CACTCGGATAAGATGCTGA**
**Hif-1** ***α***	**NM_001530**	**578**	**CTCGGCGAAGCAAAGAGT**	**CAAGCACGTCATGGGTGG**
***β*** **-actin**	**NM_001101**	**397**	**CCCGCCGCCAGCTCACCATGG**	**AAGGTCTCAAACATGATCTGGGTC**

### Western Blot Analysis

Western analysis was performed as described [49]. Protein was extracted from either whole cell lysates or nuclear and cytoplasmic fractions (NE-PER Nuclear and Cytoplasmic Extraction Reagent Kit; Pierce). Lysate protein concentrations were assayed using the Pierce BCA^®^ Protein assay. The following primary antibodies were used: rabbit anti-human eIF3a antibody (Santa Cruz Biotechnology (Cat. # sc-30149; 1∶5,000); rabbit anti-human p27^kip1^ antibody (Cell Signaling Technology, Cat. # 3698; 1∶1,000); and goat anti-human NDRG1 antibody (Abcam, Cat. # ab37897; 1∶4,000). Optimization of blotting conditions was performed using a range of antibody concentrations. The secondary antibodies used (1∶10,000) were horseradish peroxidase-conjugated anti-rabbit (eIF3a and p27^kip1^; Cat. # A0545, Sigma-Aldrich) and anti-goat (NDRG1; Cat. # A5420, Sigma-Aldrich). Chemiluminescence was performed within the linear range of the film and the substrate was not limiting. Densitometric quantitation of results was performed using BioRad Quantity One software. To ensure equal loading of proteins, membranes were probed for *β*-actin.

### Cell Migration Assay

Cell invasion was assessed using the CytoSelect™ 24-Well Cell Migration Assay (Cell Biolabs), as per the manufacturer’s protocol. Briefly, media containing 10% FCS was added to each lower well. A cell suspension (0.5×10^5^ cells/mL) in serum-free media was seeded in each insert. The plate was then incubated at 37°C/24 h. After this incubation, each insert was swabbed to remove non-migratory cells and then inserted into a clean well containing cell staining solution and incubated for 10 min at room temperature. Stained inserts were then washed with water and allowed to air dry. A photograph of cells that had migrated through the insert was taken using a light microscope (Nikon) and the inserts placed in new wells containing extraction solution. This was incubated for 10 min at room temperature on an orbital shaker. Finally, lysate was then transferred into a 96-well plate and the absorbance recorded at 560 nm on a Victor^3^ Multilabel Counter plate reader (Perkin Elmer).

### Cell Proliferation Assay

The MTT proliferation assay was used to assess cell growth rate [43]. The assay was validated by manual cell counts using Trypan blue [43].

### Cell Invasion Assay

Cell invasion was assessed using the CytoSelect™ 96-Well Cell Invasion Assay Kit (Cell Biolabs) as per the manufacturer’s protocol. Briefly, serum-free media was added to each well to rehydrate the basement membrane layer. Then, media containing 10% FCS was added to the wells of the feeder tray. A cell suspension (1×10^6^ cells/mL) in serum-free media was seeded into the membrane chamber. The plate was then incubated for 24 h/37°C. After incubation, the membrane chamber was placed into the cell harvesting tray containing “cell detachment solution” and incubated for 30 min/37°C. Subsequently, 4× Lysis Buffer/CyQuant^®^ GR dye solution was added to each well and incubated for 20 min at room temperature. Finally, the mixture was transferred to a 96-well plate and fluorescence measured at 480 nm/520 nm.

### Statistical Analysis

Results are expressed as mean ± standard deviation (SD). All experiments were performed 3–8 times and were compared using the Student’s *t*-test. Data were considered statistically significant when *p*<0.05.

## Results

### eIF3a is Recruited to Stress Granules During Iron Depletion

**Figure 1:**
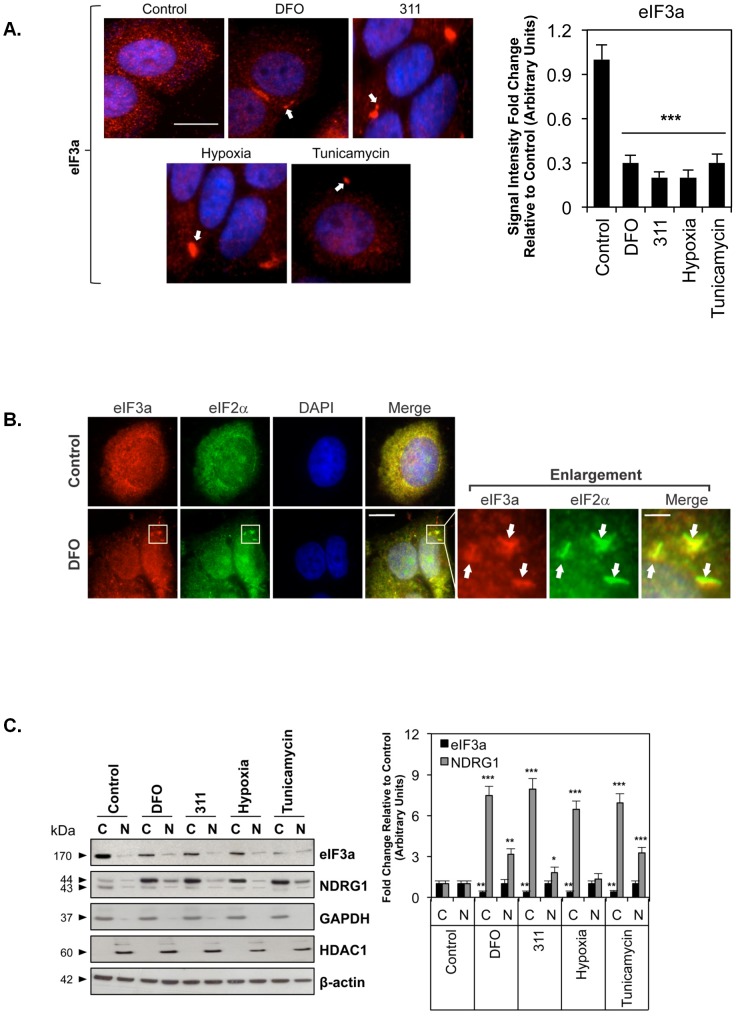
The distribution of eIF3a and NDRG1 after incubation of MCF7 cells with DFO (250 µM), 311 (25 µM), tunicamycin (5 µg/mL) or hypoxia (1% O_2_). (A) Cells were incubated using these conditions for 24 h/37°C and then stained with primary antibody against eIF3a (Alexa Fluor 555; “red”) and DAPI. Scale bar: 10 µm. (B) Co-localization of the stress granule markers eIF3a and eIF2*α* (Alexa Fluor 488; “green”) in structures consistent with stress granules after incubation with DFO under the conditions used in (A). The enlarged views of the boxed region in the merge panel are displayed to the right of this panel and show separate views for the red and green channels of the same field in which there is a cluster of eIF3a- and eIF2*α*-positive stress granules. The white arrows point to these structures. The scale bar in the left-most merge image represents 10 µm, while the scale bar in the enlarged merge image represents 2 µm. (C) Fractionation of MCF7 cells followed by western analysis demonstrated the presence of eIF3a and NDRG1 in both the cytoplasm and nucleus. GAPDH and HDAC1 were used as positive and loading controls for isolation of cytoplasmic (C) and nuclear (N) fractions, respectively. *β*-actin was used as general protein loading control. The blots are representative of 3 experiments and the densitometric analysis is expressed as mean ± SD. *p<0.05, **p<0.01, ***p<0.001 relative to the control of the same fraction.

**Figure 2:**
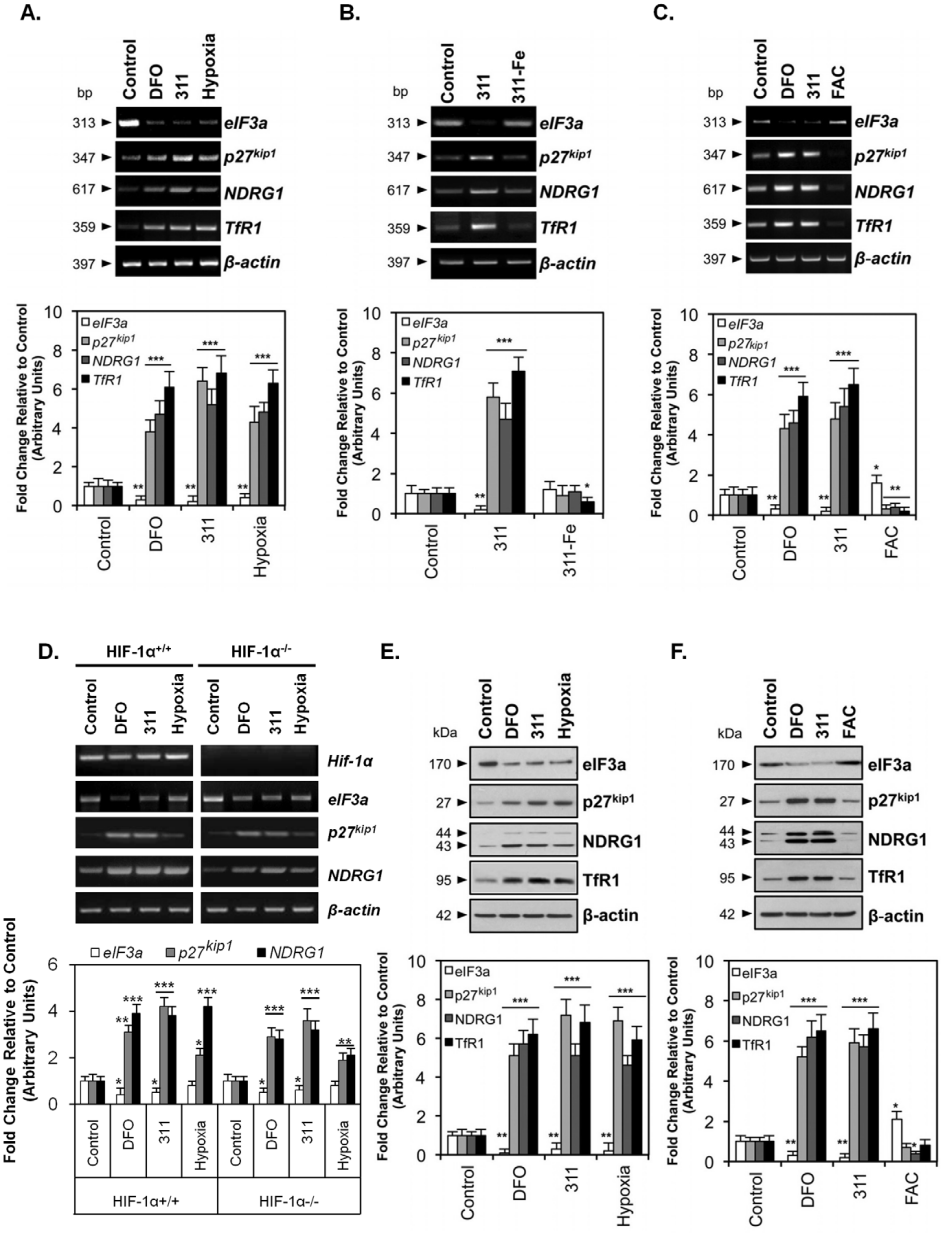
The expression of eIF3a, p27^kip1^, NDRG1 and TfR1 mRNA and protein is regulated in response to cellular iron depletion using iron chelators and also hypoxia. (A) Following incubation of MCF7 cells with DFO (250 µM), 311 (25 µM) or hypoxia (1% O_2_) for 24 h/37°C, total mRNA was extracted and RT-PCR was conducted. (B) MCF7 cells were incubated for 24 h/37°C with 311 or the pre-formed 311-iron complex (311-Fe; 2∶1 ligand-metal complex; 25 µM 311∶ 12.5 µM Fe) and total mRNA was extracted and RT-PCR conducted. (C) MCF7 cells incubated for 24 h/37°C with iron as ferric ammonium citrate (FAC; 100 µg/mL) showed an opposite expression pattern when compared to cells incubated with DFO (250 µM) for 311 (25 µM). (D) Wild-type (*HIF-1α* ^+/+^) and *HIF-1α*–knockout (KO; *HIF-1α* ^-/-^) murine embryo fibroblasts (MEFs) were incubated with either control medium, DFO (250 µM), 311 (25 µM), or hypoxia (1% O_2_) and their mRNA extracted for analysis using RT-PCR. (E) Following incubation of MCF7 cells for 24 h/37°C with the same conditions as in (A) above, total protein was extracted and western blot analysis was conducted. (F) MCF7 cells were incubated under the same conditions as in (C) and western analysis performed as in (E). The gel photographs and blots are representative of 3 independent experiments and the densitometric analysis is expressed as mean ± SD. *p<0.05, **p<0.01, ***p<0.001 relative to control cells.

The plant non-protein amino acid, l-mimosine, may act as an iron chelator to down-regulate eIF3a by an as yet unknown mechanism [7]. As l-mimosine may have pleiotropic cellular effects unrelated to its iron chelating activity, we first assessed whether eIF3a depletion and its subcellular localization were affected by classical iron depleting agents. This was achieved by incubating MCF7 cells with two well-characterized iron chelators, DFO (250 µM) or 311 (25 µM), for 24 h/37°C [39], [42]. This was then followed by analysis of eIF3a by immunofluorescence (Figure 1), RT-PCR (Figure 2) and western blotting (Figures 1 and 2). Under these conditions, DFO and 311 effectively induce cellular iron depletion in MCF7 cells and a range of other cell-types [39], [42]–[45].

Previous studies have demonstrated that eIF3a is a marker for stress granule formation, which can be readily detected in these structures by immunofluorescence analysis [16], [17]. To assess the effect of the chelators on eIF3a expression and stress granule formation, MCF7 cells were incubated with either control medium, DFO or 311, as described above. Additionally, cells were incubated with tunicamycin (5 µg/mL) or under hypoxia (1% O_2_) over the same period as additional positive controls for stress granule formation [15], [50]. The cells were then fixed and eIF3a immunofluorescence assessed (Figure 1A, B).

In control cells, eIF3a appeared as a fine red punctate pattern throughout the cytoplasm and nucleus (Figure 1A). After incubation with the agents above, the distribution of eIF3a changed and was found not only as punctate staining, but also appeared as larger discrete cytoplasmic structures ranging in size from ∼1–5 µm (see arrows - Figure 1A, B). The size of these structures is consistent with stress granules induced by various stressors [15]. Moreover, total immunofluorescence levels indicated that there was a significant (*p*<0.001) overall reduction of eIF3a expression after incubation of cells with the above iron chelators and other stressors (Figure 1A). The down-regulation of eIF3a was consistent with the adaptation of cells to stress and has been observed after incubation with l-mimosine [6], [7]. Interestingly, despite decreased eIF3a expression upon induction of stress, this molecule is known to regulate other transcripts (*e.g.*, p27^kip1^, tyrosinated *α*-tubulin and ribonucleotide reductase M2 [6], [7]). In fact, while the decrease in eIF3a is characteristic of the suppression of global translation during cellular stress, eIF3a effective regulates a subset of transcripts under these conditions [6], [7], [13], [51].

As a further control for the formation of stress granules, we performed co-localization experiments with eIF3a and another known stress granule marker, eIF2*α* [52], [53]. These studies demonstrated that both eIF2*α* and eIF3a co-localized in ∼1–5 µm structures (Figure 1B) following incubation with DFO. These data strongly suggest that cellular iron depletion induces stress granule formation. Moreover, these findings are consistent with the observation that iron depletion by l-mimosine [6], [7], as well as DFO and 311 [45], causes a suppression of global protein synthesis that is observed under stress conditions [15]. Collectively, this evidence, in combination with the fact that: ***(i)*** eIF3a is a classical marker of stress granules [16], [17]; ***(ii)*** that these structures were also identified after exposure to conditions well known to induce their formation [*i.e.*, hypoxia and tunicamycin [15], [50]; Figure 1A]; and ***(iii)*** the size of these structures was typical of stress granules [15], indicates that cellular iron depletion induces stress granule formation.

### Iron Depletion, Hypoxia and Tunicamycin Regulate eIF3a in the Cytoplasm, but not the Nucleus

The immunofluorescence images in Figure 1A represent Z-stack cross sections through nuclei indicating that the eIF3a aggregates are present in the cytoplasm and to a lesser extent in the nucleus. Cellular fractionation into cytoplasmic (C) and nuclear (N) fractions, followed by western analysis, confirmed the immunofluorescence results for eIF3a, indicating predominant cytoplasmic expression (Figure 1C). Incubation with chelators, hypoxia or tunicamycin decreased eIF3a expression in the cytoplasmic fraction relative to the control, while leaving its level in the nuclear fraction largely unaltered. The same incubation conditions increased the expression of the iron-regulated protein, NDRG1 [35]–[37], in both fractions relative to the appropriate control, strongly suggesting that the chelators induced cellular iron depletion (Figure 1C), as shown previously [39], [42]–[45]. Significantly, earlier studies have also shown that NDRG1 is localized in both the nucleus and cytoplasm [54], [55]. As shown previously [36], [37], [56], two NDRG1 bands were observed (43- and 44-kDa; Figure 1C), which may represent different post-translational modifications [56]. Thus, the densitometry for NDRG1 presented in this report represents the sum of both NDRG1 bands. It should be noted that GAPDH and histone deacetylase 1 (HDAC1) expression levels were used as controls to demonstrate fraction identity as cytoplasmic or nuclear, respectively (Figure 1C).

Taken together, these data demonstrate that different stressors (*viz*. iron depletion, hypoxia and tunicamycin) cause a similar stress response involving the formation of eIF3a-positive stress granules and the down-regulation of eIF3a expression.

### Iron Regulates eIF3a and NDRG1 mRNA Levels

We further examined the role of iron depletion in regulating eIF3a and NDRG1 expression. In good agreement with the results presented in Figure 1A, incubation of MCF7 cells with DFO or 311 resulted in a marked and significant decrease (*p*<0.01) in *eIF3a* mRNA expression compared to cells grown in control medium (Figure 2A). The influence of hypoxia (1% O_2_) was also assessed as the down-regulation of *eIF3a* expression by iron chelation may be mediated through inhibition of iron-requiring hydroxylases, which would lead to increased HIF-1*α* expression [57]. Similarly to the results with chelators, hypoxia significantly (*p*<0.01) decreased *eIF3a* mRNA expression (Figure 2A), which is consistent with a role for HIF-1*α* in the iron chelator-mediated effects observed. As positive controls, we also examined the effect of iron depletion and hypoxia on *p27* ^kip1^, *NDRG1* and *TfR1* mRNA levels, which are well-described to be increased after iron-chelation and hypoxia [35], [42], [58]–[61]. Indeed, transcript levels for all three genes were significantly (*p*<0.001) up-regulated by DFO, 311 and hypoxia relative to the control (Figure 2A).

To further assess the role of iron depletion on the down-regulation of *eIF3a* mRNA, cells were incubated with 311 (25 µM) or 311 pre-saturated with iron to form its iron complex (311-Fe; 2∶1 complex; 25 µM ligand: 12.5 µM Fe), the latter of which cannot induce iron depletion [39], [44]. In contrast to 311 which markedly and significantly (*p*<0.01) decreased *eIF3a* mRNA expression, the respective 311-iron complex had no significant (*p*>0.05) effect relative to the control (Figure 2B). A similar lack of effect of 311-Fe on increasing *p27* ^kip1^, *NDRG1* and *TfR1* mRNA expression was also demonstrated (Figure 2B). Of note, 311-Fe reduced *TfR1* mRNA relative to the control, which may indicate some donation of iron to cellular pools: an effect that has been observed with other aroylhydrazone iron complexes [62].

We next assessed the effect of the cellular iron donor, ferric ammonium citrate (FAC) [35], [63], [64], compared to chelators on *eIF3a* mRNA levels in MCF7 cells (Figure 2C). In these experiments, cells were incubated for 24 h/37°C with control medium; DFO (250 µM) or 311 (25 µM) to induce iron depletion; or with FAC to load cells with iron (100 µg/mL). As shown in Figure 2C, the expression of *eIF3a* was significantly (*p*<0.05) up-regulated in cells incubated with FAC relative to either the control or to cells incubated with iron chelators. In contrast, incubation with FAC led to a marked and significant (*p*<0.01) decrease in *p27* ^kip1^, *NDRG1* and *TfR1* mRNA expression relative to the respective controls (Figure 2C). The latter results are consistent with previous investigations demonstrating that FAC donates iron to cellular iron pools leading to down-regulation of these mRNAs [35], [65]. Collectively, these data demonstrate that intracellular iron levels modulate *eIF3a* mRNA expression, leading to its down-regulation by chelator-mediated iron depletion.

### Iron Depletion Regulates eIF3a and NDRG1 mRNAs, in part, via a HIF-1α-independent mechanism

Given our observations that *eIF3a* mRNA is down-regulated in response to iron chelation and hypoxia (Figures 2A–C), and evidence from the literature indicating *eIF3a* expression is regulated by hypoxia [18], we next examined if the *eIF3a* promoter contained the hypoxia response element (HRE) sequence (G/C/T ACGTGC G/C) that would enable regulation by HIF-1*α* [66]. However, bioinformatic analysis using the Genomatix program (Suite 3; Munich, Germany) indicated that the HRE was absent.

Nevertheless, to experimentally assess the possible involvement of HIF-1*α* in regulating *eIF3a* mRNA expression, we then compared the response of *eIF3a*, *p27^kip1^* and *NDRG1* mRNAs to iron depletion in HIF-1*α* knockout (*HIF-1α^-/-^*) MEFs to their wild-type counterparts [35], [67] (*HIF-1α^+/+^*; Figure 2D). As expected, *HIF-1α* mRNA was only detected in *HIF-1α* ^+/+^ cells (Figure 2D), as shown previously [35]. Interestingly, a considerable component of the chelator-dependent regulation of *eIF3a*, *p27^kip1^* and *NDRG1* expression in these cells was shown to be independent of HIF-1*α* (Figure 2D). Notably, the expression of *NDRG1* mRNA was significantly (*p*<0.01) higher in *HIF-1α^+/+^* cells when compared to *HIF-1α* ^-/-^ cells under all conditions (Figure 2D). These data are consistent with previous findings that HIF-1*α* plays a role in up-regulating *NDRG1* expression in response to iron depletion [35], [68].

Taken together, although HIF-1*α* appears not to be essential for the regulation of *eIF3a* or *NDRG1* mRNA by iron depletion, this transcription factor is required for mediating maximal up-regulation of NDRG1 under these conditions.

### Iron Regulates eIF3a and NDRG1 Expression at the Protein Level

Regarding the role of iron in regulating eIF3a expression at the protein level, western blot analysis (Figure 2E, F) confirmed the immunofluorescence (Figure 1A) and RT-PCR results (Figure 2A–C). As shown in Figure 2E, incubation of cells with DFO (250 µM), 311 (25 µM) or hypoxia (1% O_2_) induced a marked and significant (*p*<0.01) down-regulation of eIF3a relative to control medium alone. These experiments also confirmed that the expression of p27^kip1^, NDRG1 and TfR1 was significantly (*p*<0.001) elevated in cells exposed to either DFO, 311 or hypoxia, as shown previously [35], [60]. Moreover, in accordance with our results at the mRNA level (Figure 2A–C), western blot analysis confirmed that the expression of eIF3a, p27^kip1^ and NDRG1 at the protein level was modulated by intracellular iron levels (Figure 2F). Collectively, these results indicate that during iron depletion: ***(i)*** eIF3a mRNA and protein are down-regulated; ***(ii)*** a considerable component of the down-regulation of eIF3a mRNA is independent of HIF-1*α*; and ***(iii)*** NDRG1 and p27^kip1^ mRNA and protein levels are up-regulated.

### eIF3a Positively Regulates NDRG1 mRNA during Iron Depletion

**Figure 3:**
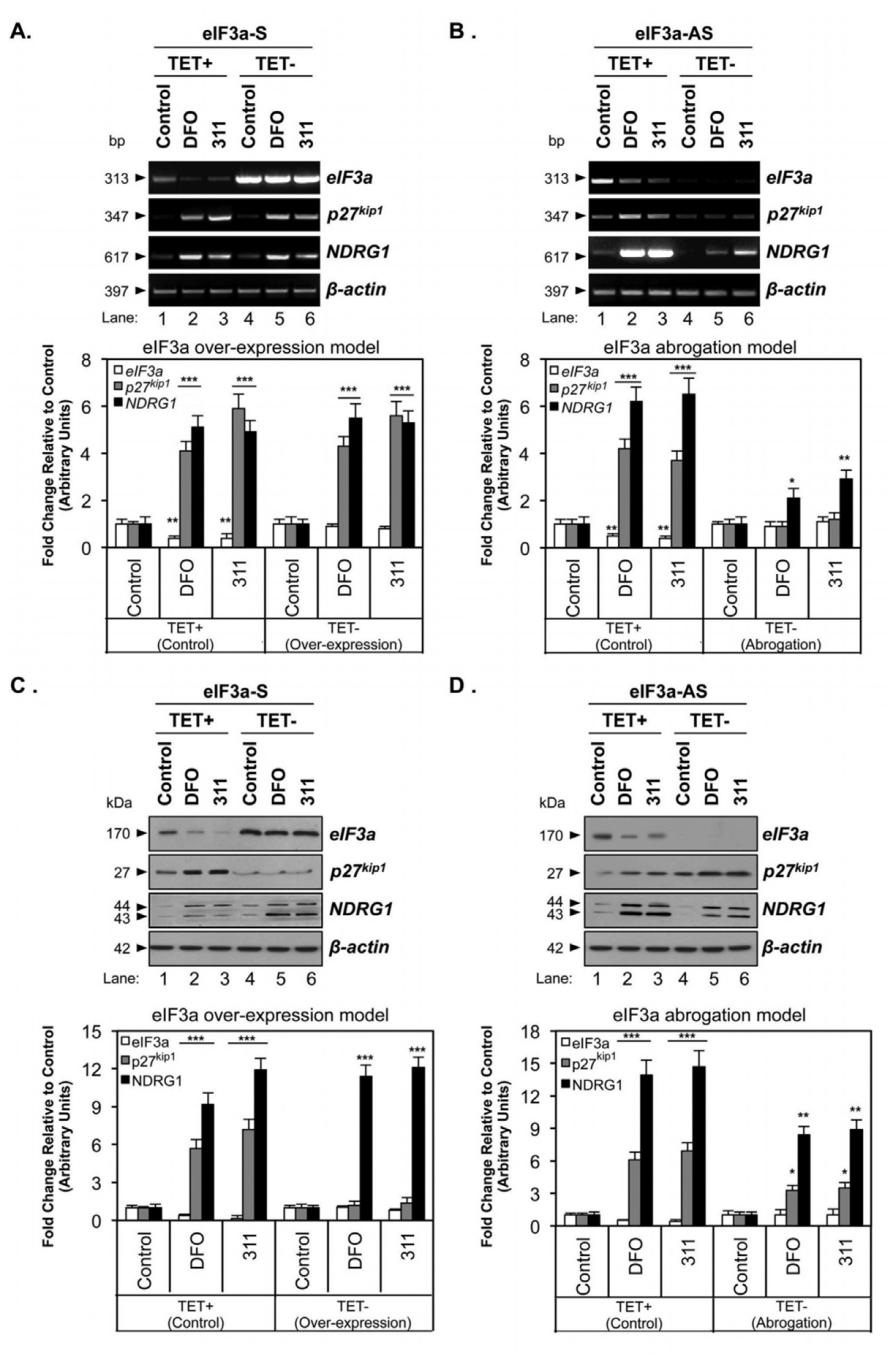
Role of eIF3a in the regulation of *p27^kip1^* and *NDRG1* mRNA and protein expression . Tetracycline (TET)-off MCF7 cells stably transfected with the eIF3a-sense plasmid (eIF3a-S; **A, C**) or the eIF3a-antisense (eIF3a-AS; **B, D**) plasmid were incubated with or without TET in either control medium, or this medium containing DFO (250 µM) or 311 (25 µM) for 24 h/37°C and their mRNA or protein extracted for analysis using RT-PCR or western blot. The gel photographs are representative of 3 independent experiments and the densitometric analysis is expressed as mean ± SD. *p<0.05, **p<0.01, ***p<0.001 relative to the respective control.

Since NDRG1 expression is regulated by iron [35], [69] and is a potent metastasis suppressor [26]–[29], we examined if eIF3a was involved in its regulation. In order to do this, a tetracycline (TET)-regulated cell-system was implemented [70]. Briefly, stably transfected TET-off MCF7 cells were transfected with a plasmid vector containing a TET-regulated element (TRE) in which either the full-length *eIF3a* sense (*eIF3a-S*) or *eIF3a* anti-sense (*eIF3a-AS*) sequences [7] were cloned. Transfected cells were selected with hygromycin (200 µg/mL) and resistant clones were verified for their ability to respond to TET. In particular, when TET was removed, the TRE was activated leading to the expression of *eIF3a-S* (Figure 3A, C, lanes 4–6) or *eIF3a-AS* (Figure 3B, D, lanes 4–6).

Using these cells, the ability of DFO or 311 to down-regulate *eIF3a* mRNA was only observed under conditions of endogenous *eIF3a* expression (*i.e.*, TET+; Figure 3A, B, lanes 1–3). The up-regulation of *eIF3a* expression in *eIF3a-S*-transfected cells by the removal of TET induced high levels of exogenous *eIF3a* mRNA (Figure 3A, lanes 4-6) and protein (Figure 3C, lanes 4–6) expression, which were insensitive to DFO or 311. Interestingly, the up-regulation of either *p27^kip1^* or *NDRG1* mRNA levels by DFO or 311 was not significantly affected by the increased expression of exogenous *eIF3a* (*i.e.*, TET-; Figure 3A, lanes 4–6) relative to the endogenous *eIF3a*-expressing (*i.e.*, TET+) samples (Figure 3A, lanes 1–3).

Conversely, when the effects of *eIF3a* mRNA ablation were examined, the expression of *eIF3a-AS* mRNA abrogated endogenous *eIF3a* mRNA (Figure 3B, lanes 4–6) and protein (Figure 3D, lanes 4–6) levels. Moreover, ablation of *eIF3a* mRNA also caused a significant (*p*<0.001) decrease in *p27^kip1^* and *NDRG1* mRNA following incubation with DFO and 311 (Figure 3B, lanes 4–6) relative to cells expressing endogenous *eIF3a* (Figure 3B, lanes 1–3). These results indicate that other than *p27^kip1^*, eIF3a also contributes to the regulation of *NDRG1* during iron depletion. It should be noted that unlike *p27^kip1^*, *NDRG1* mRNA could still be up-regulated by iron depletion when eIF3a was ablated (Figure 3B, lanes 4–6), albeit to a far lesser extent than when endogenous levels of eIF3a were expressed (Figure 3B, lanes 1–3). This result may be explained by the hypothesis that eIF3a, in addition to acting as a translational regulator, can modulate the stability of specific transcripts (*e.g.*, *p27^kip1^* and *NDRG1*). Although this possibility has yet to be experimentally tested, it is at least consistent with eIF3a’s role as a stress granule constituent and that stress granules form after incubation with DFO or 311 (Figure 1A, B). Indeed, stress granules are known to regulate mRNA stability [19].

### Effect of eIF3a Over-expression on NDRG1 and p27^kip1^ Protein Levels Under Iron Depletion

To assess if the regulation of *eIF3a*, *p27^kip1^*and *NDRG1* mRNA in the above cellular models was reflected at the protein level, western blot analysis was performed (Figure 3C, D). In cells expressing endogenous eIF3a, this protein was significantly (*p*<0.001) down-regulated upon incubation with DFO or 311 relative to the control (Figure 3C, lanes 1–3). In contrast, eIF3a levels were not regulated by iron depletion in cells over-expressing eIF3a (Figure 3C, lanes 4–6), which is in accordance with the lack of effect of iron depletion on *eIF3a* mRNA in these cells (*cf*. Figure 3A, lanes 4–6).

It is notable that the iron chelators had no effect on expression levels of exogenous *eIF3a* mRNA derived from the *eIF3a-S* cDNA-containing plasmid (Figure 3A, lanes 4–6 *vs.* 1–3). This can be rationalized by considering that expression of the cDNA from the *eIF3a-S* plasmid is regulated only by the TRE, in which transcription of the cDNA is activated by the removal of TET, and not by the native regulatory elements that would be present in the un-translated regions of the endogenous gene.

As demonstrated by Dong and Zhang [7], p27^kip1^ is down-regulated by eIF3a over-expression, which occurs predominantly at the level of translation. This finding agrees with our observation that p27^kip1^ protein levels were significantly (*p*<0.001, densitometry not shown) decreased by eIF3a over-expression (Figure 3C, *cf.* lanes 4–6 *vs*. 1–3). The down-regulation of p27^kip1^ protein occurred despite the lack of a corresponding decrease in *p27^kip1^* mRNA under these conditions (*cf*. Figure 3A, lanes 4-6 *vs*. 1–3). Furthermore, we observed that, unlike endogenous eIF3a-expressing cells (Figure 3C, lanes 1–3), p27^kip1^ levels were insensitive to iron depletion in eIF3a-over-expressing cells (Figure 3C, lanes 4–6). This insensitivity is most likely due to the observation that treatment of cells over-expressing eIF3a with iron chelators had no effect on eIF3a protein levels (Figure 3C, lanes 4–6). Collectively, these data are consistent with a translational repressor function for eIF3a on p27^kip1^ expression [7].

In contrast to the behavior of p27^kip1^, the treatment of *eIF3a-S*-transfected cells (in the presence or absence of TET) with iron chelators significantly (*p*<0.001) up-regulated NDRG1 expression (Figure 3C). Importantly, when eIF3a was over-expressed in these cells (*i.e.*, TET- condition, Figure 3C, lanes 4–6), there was a significant (*p*<0.001, densitometry not shown) ∼5-fold increase in NDRG1 in iron-depleted cells compared to the endogenous eIF3a-expressing cells (*i.e.*, TET+ condition). The increase in NDRG1 protein caused by iron depletion in eIF3a-over-expressing cells (Figure 3C, lanes 4-6) occurred despite the lack of a corresponding increase in *NDRG1* mRNA under these conditions (Figure 3A, lanes 4–6). Thus, our data suggest that the up-regulation of NDRG1 protein by eIF3a occurs *via* a translational mechanism, rather than enhancement of transcription or transcript stability. This proposal is analogous to the eIF3a-dependent up-regulation of tyrosinated *α*-tubulin and ribonucleotide reductase M2 by a translational mechanism [6], [7].

The greatest effect of eIF3a over-expression on NDRG1 expression occurred in cells that had been treated with iron chelators (Figure 3C, lanes 5,6). However, the over-expression of eIF3a still significantly (*p*<0.05; densitometry not shown) potentiated NDRG1 expression, albeit to a lesser degree, under control conditions (*i.e.*, in the absence of iron chelators) relative to cells with only endogenous eIF3a (Figure 3C, lane 4 *vs*. 1). The ability of eIF3a to up-regulate NDRG1 expression more efficiently in the presence of chelators may be due to the capability of eIF3a to maximally regulate NDRG1 expression under stress conditions (*e.g.*, after incubation with DFO or 311; Figure 3C).

### Effect of eIF3a Ablation on NDRG1 and p27^kip1^ Protein Levels Under Iron Depletion

We next examined the effect of eIF3a ablation on p27^kip1^ and NDRG1 protein levels (Figure 3D). As anticipated from the RT-PCR results presented in Figure 3B, inducible expression of the *eIF3a-AS* cassette ablated eIF3a protein expression. Notably, when comparing the controls in the *eIF3a-AS*-transfected cells in the presence and absence of TET, p27^kip1^ protein was significantly (*p*<0.001, densitometry not shown) elevated by 7-fold in cells in the absence of eIF3a relative to cells expressing endogenous eIF3a (Figure 3D, lane 4 *vs*. 1). This result further confirms that eIF3a has a repressor role in regulating p27^kip1^ protein level in MCF7 cells, as previously reported [7].

Interestingly, while DFO or 311 significantly (*p*<0.05–0.001) increased p27^kip1^ protein with or without eIF3a ablation (Figure 3D), the relative magnitude of p27^kip1^ protein up-regulation by chelators was higher in the presence of eIF3a (Figure 3D, lanes 1–3 *vs.* lanes 4–6). Additionally, the observation that p27^kip1^ protein was still significantly (*p*<0.05) up-regulated by chelators when eIF3a was ablated (Figure 3D, lanes 4–6), without any corresponding chelator-dependent increase in *p27^kip1^* mRNA (Figure 3B, lanes 4–6), is consistent with the possibility that iron chelation can function to stabilize p27^kip1^ protein, at least under conditions of low eIF3a expression. Clearly, further experiments would be required to thoroughly test this hypothesis. These data further advocate that eIF3a functions as a translational regulator during iron depletion. A hypothesis arising from these data that is worthy of further investigation is that eIF3a may also play some role in regulating the stability of specific mRNAs (*e.g.*, *p27^kip1^* and *NDRG1*) during iron depletion.

When evaluating NDRG1 expression in *eIF3a-AS*-transfected cells, we observed that ablation of eIF3a: ***(i)*** down-regulated NDRG1 under control conditions (Figure 3D, lanes 1 *vs.* 4); and ***(ii)*** diminished the up-regulation of NDRG1 occurring after incubation with DFO or 311, relative to NDRG1 levels in cells with endogenous eIF3a levels (Figure 3D, lanes 4–6 *vs.* lanes 1–3). In fact, when eIF3a was ablated, the up-regulation of NDRG1 by chelators was only 8-9-fold greater than the respective control (Figure 3D, lanes 4–6) compared to the 14–15-fold up-regulation observed in cells with endogenous eIF3a levels (Figure 3D, lanes 1–3). Thus, the ablation of eIF3a significantly (*p*<0.01, densitometry not shown) diminished the ability of the chelators to up-regulate NDRG1.

Collectively, the results in Figures 3C, D demonstrate that eIF3a modulation alters the expression of both p27^kip1^ and NDRG1 in different ways. For example, eIF3a over-expression represses p27^kip1^ levels, while potentiating the increase in NDRG1 levels under conditions of iron depletion (Figure 3C, lanes 4–6). In contrast, eIF3a ablation up-regulates p27^kip1^, but leads to less efficient up-regulation of both NDRG1 and p27^kip1^ relative to the control by iron depletion (Figure 3D, lanes 4–6 *vs.* Figure 3D, lanes 1–3). These observations strongly suggest a role for eIF3a in differentially regulating the translation, and potentially the stability, of specific mRNAs during cellular stress caused by iron depletion [6], [7].

### Altered Migration and Invasion in TET-Regulated eIF3a-S and eIF3a-AS Cells

NDRG1 is a well characterized and potent metastasis suppressor [22], [71]. In fact, NDRG1 has been demonstrated to regulate cell differentiation, cell cycle progression [26], [27] and to markedly inhibit cellular migration and invasion [36], [37], [72]. Indeed, a general characteristic of metastatic cancer cells is the acquisition of a higher motility rate [73]. Considering eIF3a regulates NDRG1 expression (Figure 3), we examined whether modulation of eIF3a altered cell motility using cell migration and invasion assays.

**Figure 4:**
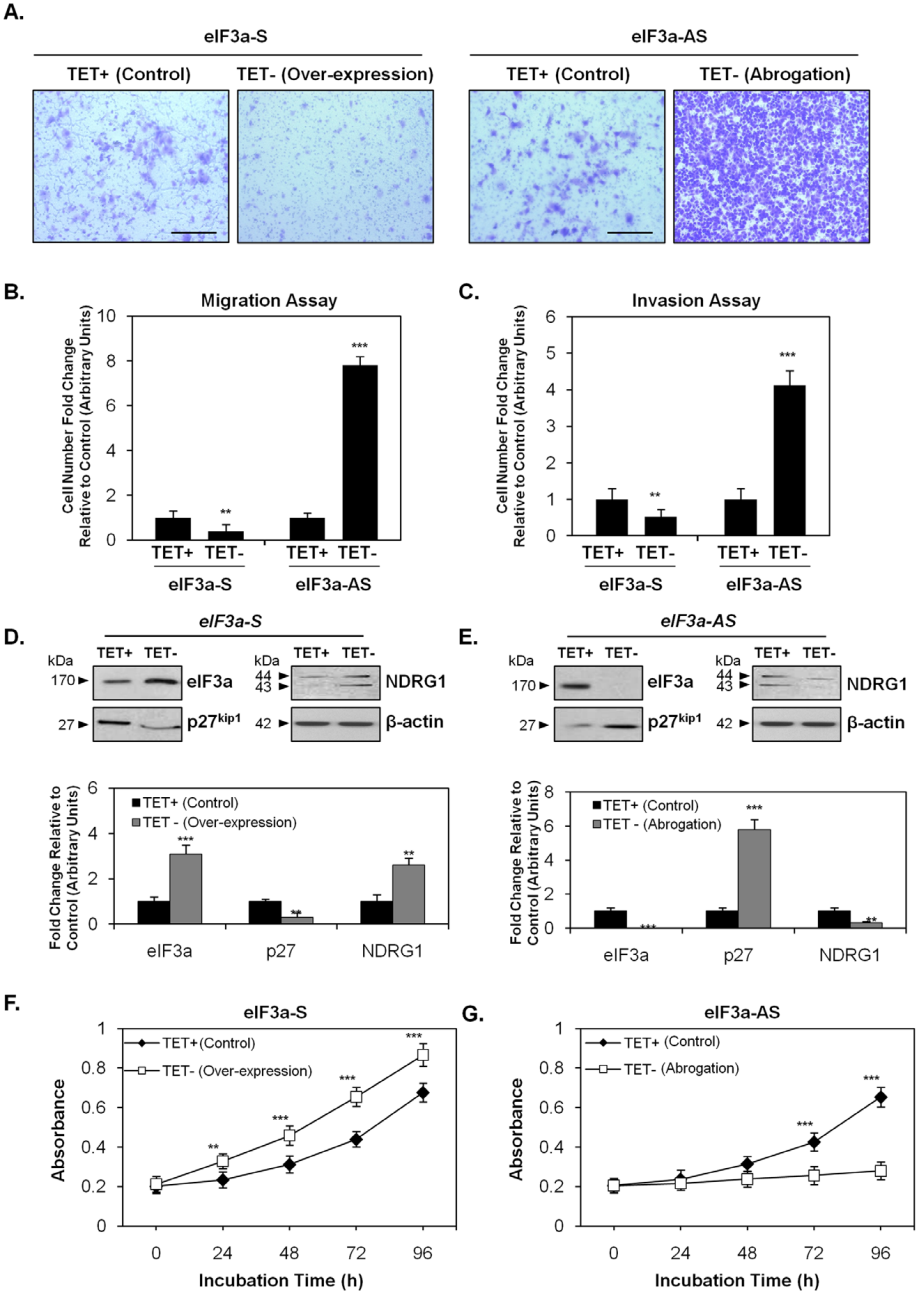
Altered cell migration, invasion and proliferation implementing TET-regulated eIF3a-S and eIF3a-AS transfected MCF7 cells. (A) The images show the result of a migration assay and were taken after a 24 h/37°C incubation of eIF3a-S and eIF3a-AS transfected MCF7 cells in the presence or absence of TET. Cell density indicates the number of cells that were able to pass through a polycarbonate membrane with a pore size of 8 µm. Scale bar: 200 µm. (B) The graph quantifies the result of the migration assays performed in (A) and is expressed as the fold change of the number of cells relative to the control. (C) The graph illustrates the result of invasion assays as the fold change in the number of cells that were able to pass through a basement membrane relative to the control. The western blots in (D) and (E) represent the expression of eIF3a, p27^kip1^ and NDRG1 under the conditions shown using eIF3a-S and eIF3a-AS cells in the presence and absence of TET. (F, G) The graphs show the proliferation of eIF3a-S and eIF3a-AS transfected MCF7 cells in the presence and absence of TET over a 24, 48, 72 and 96 h period. The photographs in (A) and western blots in (D, E) are representative of 3 experiments. Migration (B), invasion (C) and densitometry (D, E) are expressed as mean ± SD (3 experiments), while proliferation results (F, G) are expressed as mean ± SD (8 experiments). **p<0.01, ***p<0.001.

As illustrated in Figure 4A and B, under control conditions, cells with endogenous eIF3a levels (*i.e.*, *eIF3a-S* TET+ or *eIF3a-AS* TET+; see lane 1, Figs 3C, D) similarly migrated through a polycarbonate membrane (8 µm pore size) after 24 h. However, the eIF3a over-expression model (*eIF3a-S*, TET-) showed a significantly (*p*<0.01) reduced ability to migrate (Figures 4A, B). These cells also demonstrated a significantly (*p*<0.01) impaired ability to invade into an artificial basement membrane and matrix relative to endogenous eIF3a-expressing (*i.e.*, *eIF3a-S*, TET+) control cells (Figure 4C). Given that eIF3a potentiates NDRG1 expression (Figure 3C), the ability of eIF3a over-expression to down-regulate migration and invasion could be related to the significant (*p*<0.01) increase in expression of the metastasis suppressor, NDRG1, in eIF3a-over-expressing cells (Figure 4D). In contrast, when eIF3a expression was ablated using the *eIF3a-AS* cells (TET-), there was a marked and significant (*p*<0.001) ∼8-fold increase in cell migration (Figures 4A, B) and a ∼4-fold increase in cell invasion relative to *eIF3a-AS* (TET+) control cells (Figure 4C). This effect could be related to the significant (*p*<0.01) decrease in NDRG1 expression mediated by the abrogation of eIF3a (Figure 4E). Notably, when eIF3a expression was abolished [*i.e.*, *eIF3a-AS* cells (TET-)], p27^kip1^ was significantly (*p*<0.001) up-regulated (Figure 4E) which would block proliferation [74]. The increase in p27^kip1^ elicited by the ablation of eIF3a would not be expected to affect motility or invasion potential, as it is a cyclin-dependent kinase inhibitor that inhibits proliferation [74]. Indeed, we observed that motility and invasion were up-regulated under these conditions (Figures 4A–C).

These results demonstrate that the over-expression of eIF3a impairs cell motility and invasion (Figure 4A–C). Due to NDRG1’s known tumor-suppressive activity [22], the impairment of cell motility and invasion potential by eIF3a over-expression may be due to the ability of eIF3a to up-regulate the expression of NDRG1 (Figure 4D, E). In contrast, cells with ablation of eIF3a show acquired motility and invasive behavior (Figure 4A–C), which may be due to the observed down-regulation of NDRG1 in these cells (Figure 4D, E).

### Altered Proliferation in TET-Regulated eIF3a-S and eIF3a-AS Cells

In order to determine the effect of eIF3a expression on alterations in cellular growth, proliferation assays were also performed. Figure 4F shows the results of these assays for MCF7 cells transfected with the *eIF3a-S* plasmid (over-expression model; *eIF3a-S*, TET-). In these cells, the over-expression of eIF3a (Figure 4D) led to significantly (*p*<0.001-0.01) higher proliferation than in endogenous eIF3a-expressing control cells after a 24–96 h incubation (Figure 4F). Although the mechanism by which eIF3a positively regulates proliferation is unknown, the eIF3a-dependent down-regulation of the cyclin-dependent kinase inhibitor, p27^kip1^, the latter of which inhibits cellular proliferation [74], is a likely candidate.

In contrast, when eIF3a expression was ablated by expression of the *eIF3a-AS* construct, cell proliferation was markedly and significantly (*p*<0.001) reduced relative to control cells after 72 and 96 h (Figure 4G). Again, this observation is a probable consequence of the up-regulation of p27^kip1^ in the absence of eIF3a (Figure 4E).

These data above indicate the eIF3a-dependent changes in motility and invasion observed in Figure 4A–C were not simply due to corresponding alterations in cell proliferation. In fact, eIF3a regulated motility/invasion and proliferation in opposite directions, with lower motility/invasion in highly proliferating cells (*i.e.*, *eIF3a-S*, TET-) and higher motility/invasion in those cells with lower proliferation rates (*i.e.*, *eIF3a-AS*, TET-). Taken together, these results are consistent with a model in which eIF3a, through its effects on the expression of NDRG1 and p27^kip1^, plays a role in the regulation of cell migration/invasion and proliferation, respectively.

## Discussion

This is the first investigation to identify a role for eIF3a in regulating NDRG1 expression. We also demonstrated the formation of eIF3a-containing stress granules after iron depletion. The latter event was accompanied by a decrease in eIF3a expression that occurs during iron depletion with the iron chelator, l-mimosine [6], [7]. Moreover, the reduced levels of eIF3a observed after iron depletion are reflective of the cellular stress response that suppresses global protein synthesis, while allowing for translation of proteins that are vital for mounting an adequate stress response [15].

### NDRG1 Expression is Regulated by eIF3a

While iron depletion decreased eIF3a, both NDRG1 and the well characterized repressed target of eIF3a, p27^kip1^ [7], were up-regulated (Figure 2A,D). To understand the potential role of eIF3a in regulating NDRG1 mRNA and protein expression, we carried out inducible eIF3a expression or ablation studies in control or iron-depleted cells. These results indicate that eIF3a positively regulates NDRG1 expression, while it negatively regulates p27^kip1^ expression. Consistent with our findings, it is known that eIF3a positively regulates translation of other transcripts (*e.g.*, the mRNA for ribonucleotide reductase M2), despite its decreased expression during stress [6], [7]. Therefore, although iron depletion down-regulates eIF3a, this protein still potentiates NDRG1 expression under this stress condition.

**Figure 5:**
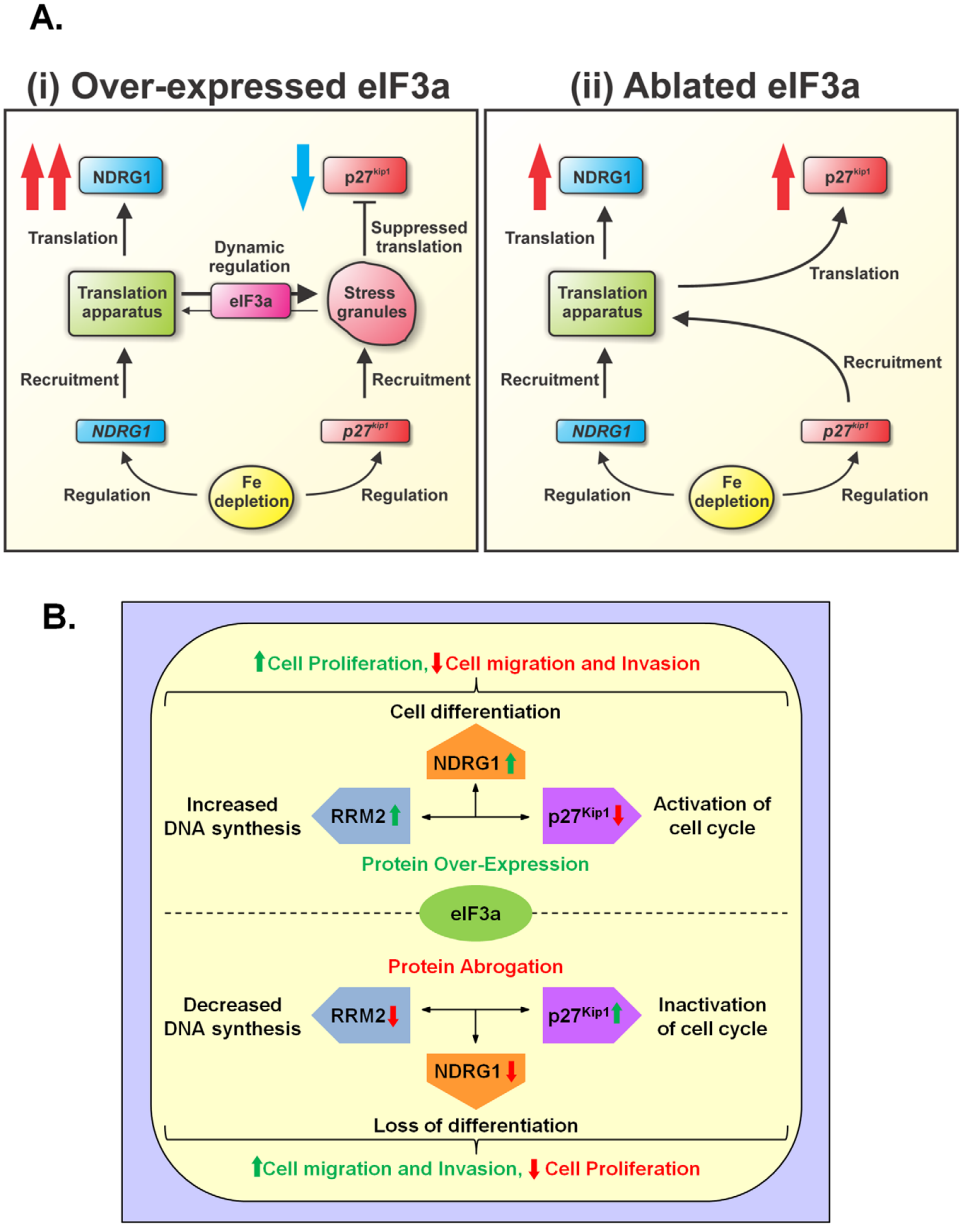
Schematic overview of the down-stream genes regulated by eIF3a and the resultant functional effects. (A) A working model that describes eIF3a’s role in regulating NDRG1 and p27^kip1^ expression. **(i)** When eIF3a is over-expressed, iron depletion up-regulates *NDRG1* transcription and eIF3a stimulates translation of nascent *NDRG1* transcripts due to its pro-translation role as an initiation factor subunit. This facilitates *de novo* NDRG1 synthesis, while translation of non-essential transcripts is suppressed during stress [15]. Conversely, our observation that p27^kip1^ protein, but not mRNA, is down-regulated by eIF3a over-expression suggests p27^kip1^ transcripts may be instead recruited to stress granules, the production of which is dynamically regulated by eIF3a, thereby suppressing p27^kip1^ synthesis. (**ii)** In contrast, when eIF3a is ablated, eIF3a-containing stress granules do not form and *p27^kip1^* transcripts are, by default, recruited by the translational apparatus, thereby increasing p27^kip1^ protein expression during iron depletion. In the absence of eIF3a, *NDRG1* transcripts continue to be directed to the translational apparatus, but are translated at a slower rate due to the loss of eIF3a. This model is consistent with the data presented in this study and with the known ability of eIF3a to negatively regulate p27^kip1^ expression by a translational mechanism [7]. **(B)** Schematic summarizing some of the functions of eIF3a, including those demonstrated in this study. First, when eIF3a is over-expressed such as in early stages of cancer [80] there is: **(i)** up-regulation of the metastasis suppressor, NDRG1, leading to both increased differentiation [22], [71] and decreased metastasis/invasion (shown herein); **(ii)** down-regulation of the cyclin-dependent kinase inhibitor, p27^kip1^, resulting in activation of the cell cycle and proliferation (shown herein and [7]); and **(iii)** increased expression of the ribonucleotide reductase M2 subunit (RRM2) [6] allowing DNA synthesis and growth. Second, when eIF3a expression is abrogated, such as occurs in hypoxic tissues that are typical of advanced tumors [77], [78], there is: **(i)** down-regulation of NDRG1, leading to a loss of differentiation and increased metastasis/invasion (shown herein); **(ii)** up-regulation of p27^kip1^, resulting in the inactivation of the cell cycle and inhibited proliferation [7]; and **(iii)** decreased expression of RRM2 [6], preventing DNA synthesis and growth.

Considering the mechanism involved in the eIF3a-dependent up-regulation of NDRG1 under stress conditions, our observations are consistent with a model in which eIF3a’s activity in regulating the balance between translation and its suppression occurs *via* the interplay between stress granules and the sites of translation [6], [7], [15], [75]. Typically, stress granules suppress translation of non-essential transcripts [75]. Thus, a working model that accounts for the findings herein is that iron depletion up-regulates *NDRG1* transcription [35], and under conditions of eIF3a over-expression, there is an eIF3a-stimulated increase in translation of nascent *NDRG1* transcripts (Figure 5Ai). This facilitates *de novo* NDRG1 synthesis, while translation of non-essential transcripts is suppressed during stress [15]. Conversely, as p27^kip1^ protein, but not mRNA, is down-regulated by eIF3a over-expression (Figure 3A, C), this suggests *p27^kip1^* transcripts are instead recruited to stress granules which are dynamically regulated by eIF3a, thereby suppressing p27^kip1^ synthesis. In contrast, when eIF3a is ablated, eIF3a-containing stress granules do not form and *p27^kip1^* transcripts are recruited by the translational apparatus, thereby increasing p27^kip1^ protein expression during iron depletion (Figure 5Aii). In the absence of eIF3a, *NDRG1* transcripts are still directed to the translational apparatus, but are translated at a slower rate due to loss of eIF3a (Figure 5Aii). This model is consistent with the data herein and with eIF3a’s ability to negatively regulate p27^kip1^ expression by a translational mechanism [7].

### eIF3a Negatively Regulates Migration and Invasion but Positively Regulates Proliferation

The increased expression of eIF3a has been documented in a wide range of cancer cell lines and tumors compared with their non-cancerous counterparts [14]. Such results have been taken to suggest that eIF3a may be involved in oncogenesis [14]. It has previously been demonstrated that eIF3a over-expression stimulates cellular proliferation and stimulates cell cycle progression [6], [9], which are key aspects of the malignant phenotype. Indeed, in the present study, we confirmed that that ablation of eIF3a suppressed proliferation, while eIF3a over-expression stimulated proliferation. However, when we examined two other key malignant properties (*e.g.*, migration and invasion potential), we observed that eIF3a ablation significantly enhanced cell migration and invasion. Conversely, the over-expression of eIF3a significantly suppressed cell migration and invasion. These results are important for understanding the role of eIF3a in cancer development and metastasis, and suggest that eIF3a may differentially regulate different aspects of malignancy.

A previous proteomic analysis [23] indicated NDRG1 was involved in the early response to the chelator, l-mimosine, which is a well known pharmacological regulator of eIF3a [7]. However, the role of eIF3a in regulating NDRG1 was not examined. Additional support for a potential relationship between eIF3a and its role in NDRG1 expression was suggested by observations that: ***(i)*** an increase in intracellular calcium was necessary for NDRG1 up-regulation [76] and ***(ii)*** that calcium was necessary for the function of eIF3a as part of the stress granule [16].

According to the theory of hypoxic tolerance, during acute hypoxia, global translation is suppressed, while the translation of a defined group of mRNAs is modulated [15]. Our results suggest that, similarly to p27^kip1^ up-regulation [7], NDRG1 levels are increased under acute hypoxia or iron-deprivation, which is probably due, in part, to the translational regulatory activity of eIF3a. This has important consequences when considering the role of eIF3a in p27^kip1^ expression [6], [7] as, following prolonged hypoxia which is often found in advanced tumors, eIF3a expression is markedly down-regulated [77], [78]. Under such conditions, which were mimicked in this study using the eIF3a abrogation model (*eIF3a-AS* cells; Figure 3D), the eIF3a-mediated up-regulation of NDRG1 would not occur, which may potentiate metastasis. Figure 5B summarizes the role of eIF3a in modulating NDRG1 and p27^kip1^ expression, which would be expected to reciprocally regulate proliferation or migration and invasion. Intriguingly, eIF3a is involved in regulating proliferation and differentiation [79], [80] and our data suggest the regulation of differentiation may be due to the modulation of NDRG1 through alteration of eIF3a expression. In fact, NDRG1 is well known to play a role in differentiation of tissues and tumors [22], [71].

Our current results are relevant to understanding the role of eIF3a in carcinogenesis. For instance, high eIF3a levels, which are typical of early cancer stages [80], increase proliferation (potentially due to low p27^kip1^ expression; Figure 4D, F) and differentiation [79], while decreasing migration and invasion (possibly due to high NDRG1 expression; Figure 4A–D). On the other hand, eIF3a abrogation, which is typical of advanced hypoxic solid cancers [77], [78], correlates with lower proliferation (potentially due to high p27^kip1^ expression; Figure 4E, G) and lower differentiation grade, while promoting acquisition of a metastatic phenotype (possibly due to lower NDRG1 expression; Figure 4E). Significantly, the differential expression of p27^kip1^ at several tumor stages has been described [81], [82], supporting this latter hypothesis.

In summary, for the first time, we demonstrate that iron depletion induces eIF3a-positive stress granules and decreases eIF3a expression, the latter of which probably occurs through stress-dependent suppression of translation. However, these low eIF3a levels during iron depletion are still effective at increasing NDRG1 and p27^kip1^. Furthermore, modulation of eIF3a expression led to altered proliferation, migration and invasion, which could depend on the concurrent regulation of both p27^kip1^ and NDRG1. Together, these results are relevant to understanding the molecular alterations involved in proliferation and metastasis.
