# Correction: N-myc Downstream Regulated 1 (NDRG1) Is Regulated by Eukaryotic Initiation Factor 3a (eIF3a) during Cellular Stress Caused by Iron Depletion

**DOI:** 10.1371/journal.pone.0149922

**Published:** 2016-02-25

**Authors:** Darius J. R. Lane, Federica Saletta, Yohan Suryo Rahmanto, Zaklina Kovacevic, Des R. Richardson

Due to a clerical error by the authors, Fig 1A of the original article includes overlapping images of the same cluster of cells in the "311" panel and in the "hypoxia" panel. In the revised version of Fig 1 provided here, all images in Fig 1A have now been replaced by replicate images obtained in parallel experiments. These replicate images show the same result as in the original Fig 1A, and demonstrate that a range of stressors (e.g., DFO, 311, hypoxia and tunicamycin) lead to the formation of eIF3a- containing structures consistent with stress-granules. The raw, uncropped immunofluorescence images for the revised figure are provided as a Supporting Information file.

**Fig 1:**
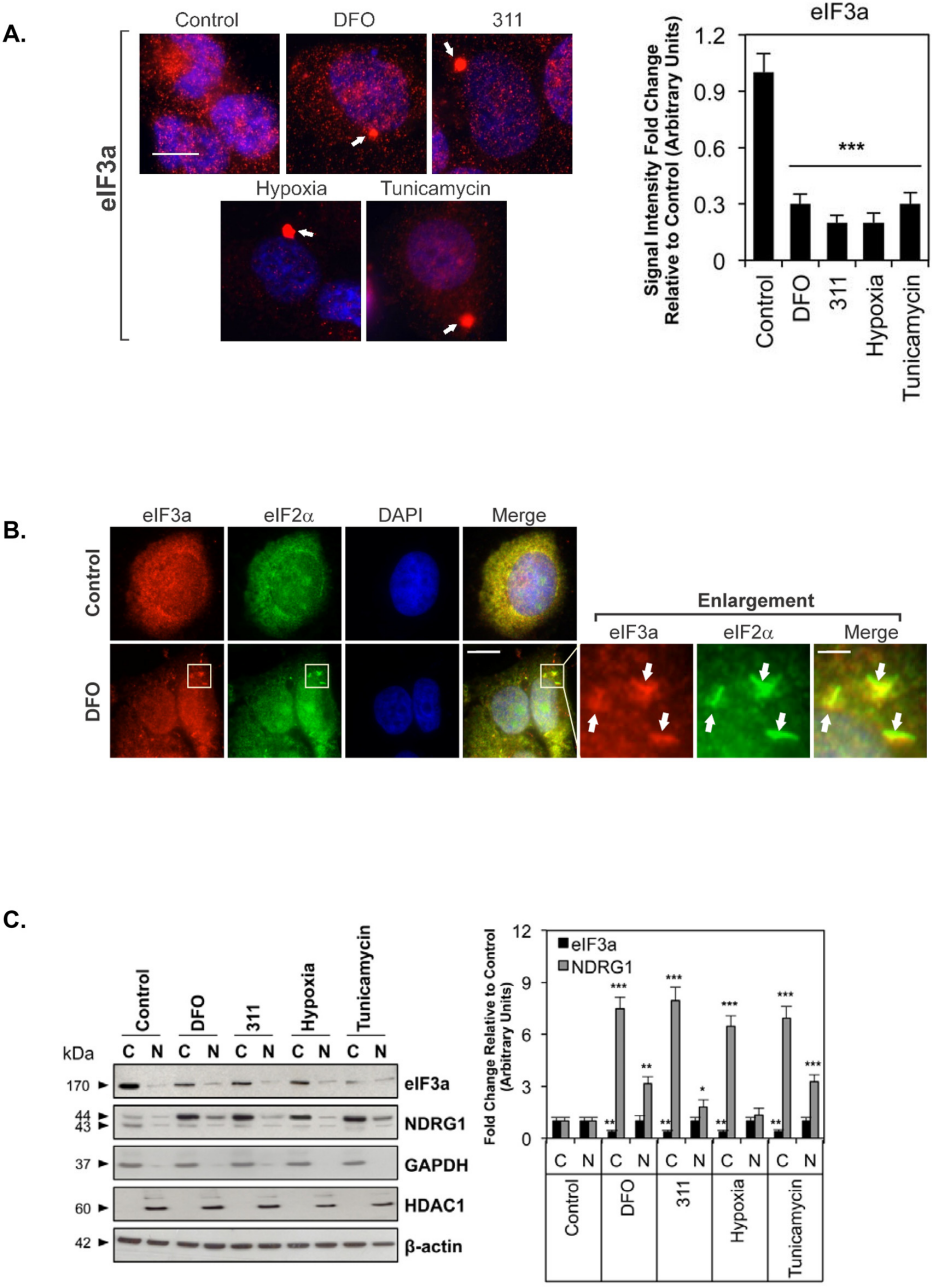
The distribution of eIF3a and NDRG1 after incubation of MCF7 cells with DFO (250 μM), 311 (25 μM), tunicamycin (5 μg/mL) or hypoxia (1% O_2_). (A) Cells were incubated using these conditions for 24 h/37°C and then stained with primary antibody against eIF3a (Alexa Fluor 555; “red”) and DAPI. Scale bar: 10 μm. (B) Co-localization of the stress granule markers eIF3a and eIF2*α*(Alexa Fluor 488; “green”) in structures consistent with stress granules after incubation with DFO under the conditions used in (A). The enlarged views of the boxed region in the merge panel are displayed to the right of this panel and show separate views for the red and green channels of the same field in which there is a cluster of eIF3a- and eIF2*α*-positive stress granules. The white arrows point to these structures. The scale bar in the left-most merge image represents 10 μm, while the scale bar in the enlarged merge image represents 2 μm. (C) Fractionation of MCF7 cells followed by western analysis demonstrated the presence of eIF3a and NDRG1 in both the cytoplasm and nucleus. GAPDH and HDAC1 were used as positive and loading controls for isolation of cytoplasmic (C) and nuclear (N) fractions, respectively. *β*-actin was used as general protein loading control. The blots are representative of 3 experiments and the densitometric analysis is expressed as mean ± SD. *p<0.05, **p<0.01, ***p<0.001 relative to the control of the same fraction.

## Supporting Information

S1 File Raw, uncropped immunofluorescence images for revised Fig 1A and 1B. (PPTX)
