# Supplementary material for: Correction: N-myc Downstream Regulated 1 (NDRG1) Is Regulated by Eukaryotic Initiation Factor 3a (eIF3a) during Cellular Stress Caused by Iron Depletion
Source: PLoS One. 2016 Feb 25;11(2):e0149922. doi: 10.1371/journal.pone.0149922 (PMC4767321; doi:10.1371/journal.pone.0149922)
Supplement: S1 File — (PPTX) [file pone.0149922.s001.pptx]

## Slide 1
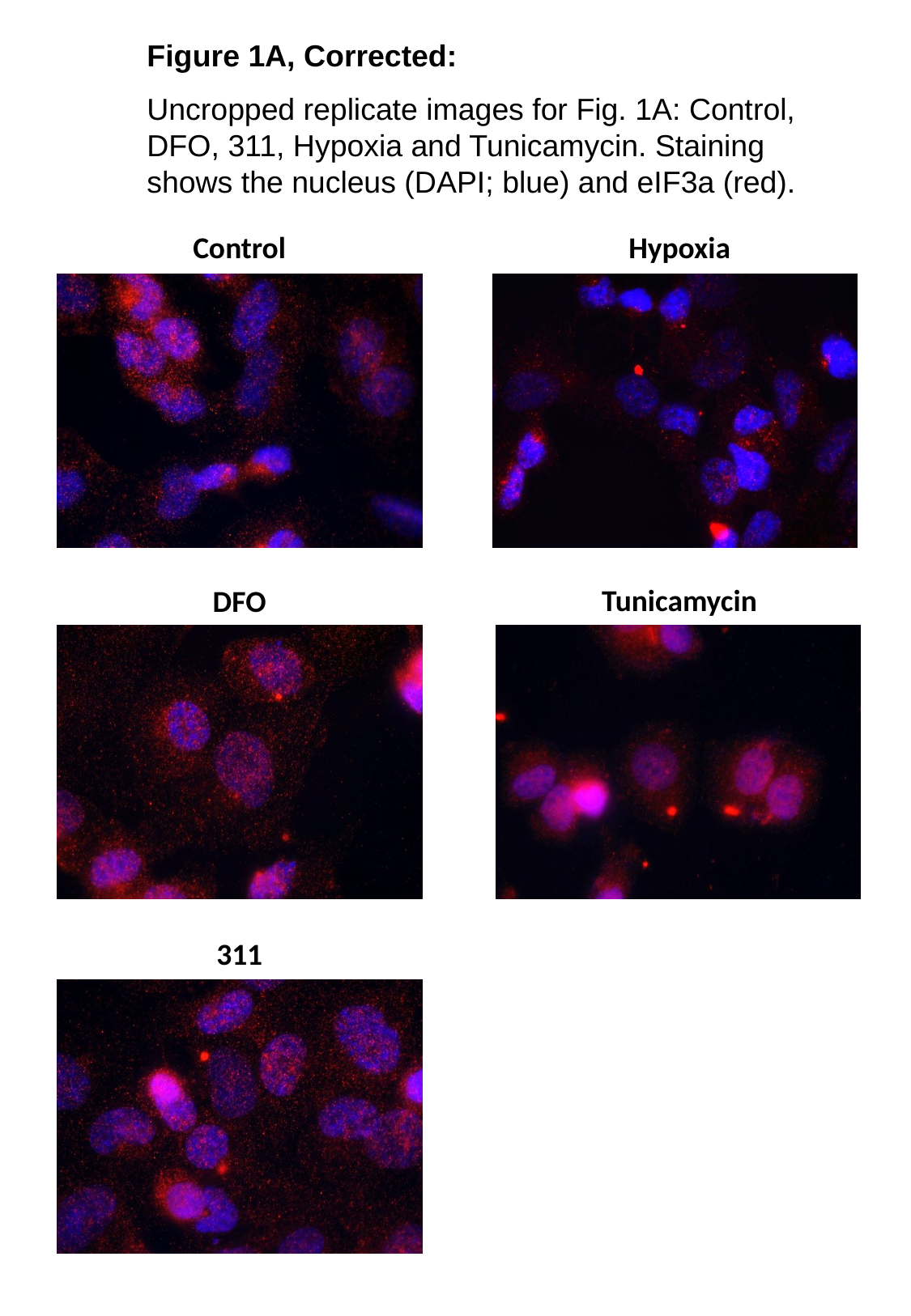

Figure 1A, Corrected:
Uncropped replicate images for Fig. 1A: Control, DFO, 311, Hypoxia and Tunicamycin. Staining shows the nucleus (DAPI; blue) and eIF3a (red).
Control
Hypoxia
Tunicamycin
DFO
311

## Slide 2
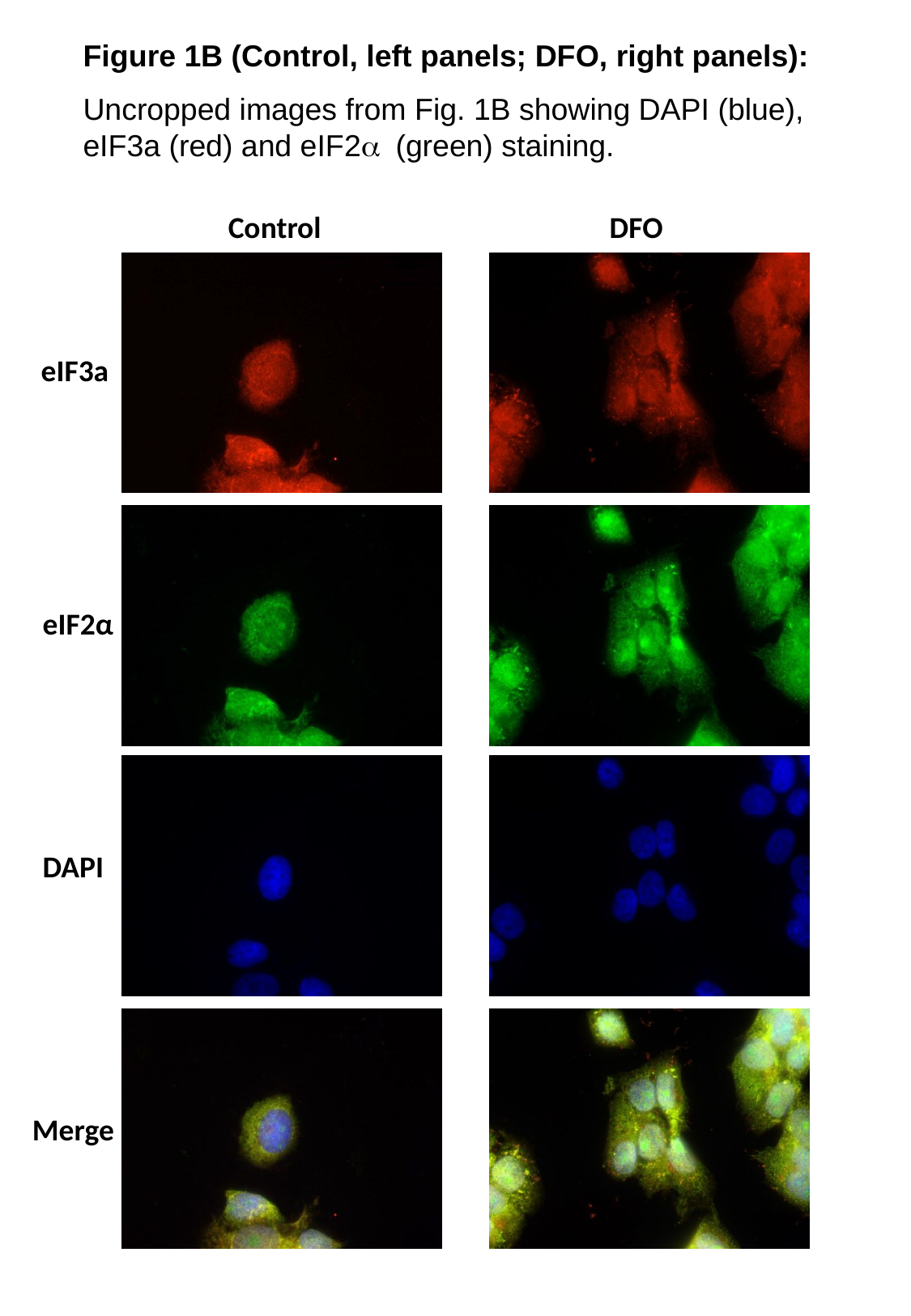

Figure 1B (Control, left panels; DFO, right panels):
Uncropped images from Fig. 1B showing DAPI (blue), eIF3a (red) and eIF2a (green) staining.
Control
DFO
eIF3a
eIF2α
DAPI
Merge
